# Altered cerebellar connectivity in Parkinson's patients ON and OFF L-DOPA medication

**DOI:** 10.3389/fnhum.2015.00214

**Published:** 2015-04-21

**Authors:** Sara B. Festini, Jessica A. Bernard, Youngbin Kwak, Scott Peltier, Nicolaas I. Bohnen, Martijn L. T. M. Müller, Praveen Dayalu, Rachael D. Seidler

**Affiliations:** ^1^Center for Vital Longevity, School of Behavioral and Brain Sciences, University of Texas at DallasDallas, TX, USA; ^2^Department of Psychology, University of MichiganAnn Arbor, MI, USA; ^3^Department of Psychology and Neuroscience, University of Colorado BoulderBoulder, CO, USA; ^4^Department of Psychological and Brain Sciences, University of Massachusetts AmherstAmherst, MA, USA; ^5^Functional MRI Laboratory, Department of Biomedical Engineering, University of MichiganAnn Arbor, MI, USA; ^6^Department of Radiology, University of MichiganAnn Arbor, MI, USA; ^7^Department of Neurology, University of MichiganAnn Arbor, MI, USA; ^8^Geriatric Research, Education and Clinical Center, VA Ann ArborAnn Arbor, MI, USA; ^9^School of Kinesiology, University of MichiganAnn Arbor, MI, USA

**Keywords:** cerebellum, resting state functional connectivity, Parkinson's disease (PD), L-DOPA, lobules

## Abstract

Although nigrostriatal changes are most commonly affiliated with Parkinson's disease, the role of the cerebellum in Parkinson's has become increasingly apparent. The present study used lobule-based cerebellar resting state functional connectivity to (1) compare cerebellar-whole brain and cerebellar-cerebellar connectivity in Parkinson's patients both ON and OFF L-DOPA medication and controls, and to (2) relate variations in cerebellar connectivity to behavioral performance. Results indicated that, when contrasted to the control group, Parkinson's patients OFF medication had increased levels of cerebellar-whole brain and cerebellar-cerebellar connectivity, whereas Parkinson's patients ON medication had decreased levels of cerebellar-whole brain and cerebellar-cerebellar connectivity. Moreover, analyses relating levels of cerebellar connectivity to behavioral measures demonstrated that, within each group, increased levels of connectivity were most often associated with improved cognitive and motor performance, but there were several instances where increased connectivity was related to poorer performance. Overall, the present study found medication-variant cerebellar connectivity in Parkinson's patients, further demonstrating cerebellar changes associated with Parkinson's disease and the moderating effects of medication.

## Introduction

Parkinson's disease is a neurodegenerative disorder characterized by both motor and cognitive impairments. While Parkinson's is most typically associated with denervation of the substantia nigra (e.g., Fearnley and Lees, 1991; Braak et al., 2006) and the resulting striatal dopamine depletion (e.g., Hornykiewicz, 1966; Jenner, 1989; Rakshi et al., 1999), the role of the cerebellum in Parkinson's disease has recently received considerable attention (for a review see Wu and Hallett, 2013).

An increasing amount of research has found distinct cerebellar changes in Parkinson's patients. Structurally, Parkinson's patients have decreased cerebellar volume compared to controls (Camicioli et al., 2009; Borghammer et al., 2010; cf. Feldmann et al., 2008), and Parkinson's patients with tremor have decreased cerebellar gray matter compared to those without tremor (Benninger et al., 2009). Functionally, when compared to controls, Parkinson's patients OFF dopaminergic medication have exhibited greater activity in the cerebellum while performing various upper limb movements (Jahanshahi et al., 1995; Catalan et al., 1999; Wu and Hallett, 2005, 2008; Cerasa et al., 2006; Yu et al., 2007; Wu et al., 2010). Greater activation of the ipsilateral cerebellar hemisphere in Parkinson's patients OFF medication has also been observed during a hand movement task, when compared to patients ON medication and controls (Rascol et al., 1997). Enhanced cerebellar activation was present during early motor learning in Parkinson's patients ON medication as well, when behavioral performance was equivalent between patients and controls (Bédard and Sanes, 2009).

Moreover, some cerebellar differences in Parkinson's patients have been attributed to potential compensatory changes. For instance, Palmer et al. (2009b) found that, when performing a sinusoidal force-tracking task at faster speeds, Parkinson's patients OFF medication uniquely activated the cerebellar hemispheres bilaterally relative to controls. This effect was partially reduced by L-DOPA medication. Further, Parkinson's patients OFF medication also exhibited significantly greater cerebellar-whole brain functional connectivity and significantly reduced striatal-cortical functional connectivity, when compared to controls, during the same force-tracking task (Palmer et al., 2010). This differentially greater cerebellar activation and cerebellar connectivity was interpreted as compensatory for three reasons. First, Parkinson's patients had similar behavioral performance as controls. Second, both the spatial extent and magnitude of the cerebellar activity increased with speed, indicating that perhaps greater cerebellar resources were needed to accommodate for greater task demands. And, third, Parkinson's patients activated the basal ganglia and thalamus less than controls, suggesting that the cerebellum may be recruited to overcome denervation of these striatal networks (see also Palmer et al., 2009a).

Here, we further probe cerebellar function in Parkinson's disease by utilizing resting state functional connectivity analyses in conjunction with motor and cognitive assessments. Resting state connectivity analyses provide insight into the underlying unconstrained functional networks of the brain, as opposed to analyses of task-based neural connectivity. Specifically, resting state functional connectivity identifies brain regions with similar time courses of spontaneous brain activity. Prior research has shown that brain regions with known anatomical connections and similar theorized functions show strong functional connectivity at rest (e.g., Fox and Raichle, 2007; Rogers et al., 2007; Vincent et al., 2007; Van Den Heuvel and Hulshoff Pol, 2010), suggesting that assessing resting connectivity has bearings on functional and anatomical relationships. Additionally, we have recently documented cerebellar connectivity differences in healthy aging (Bernard et al., 2013), providing a foundation for similar assessment in Parkinson's disease.

Specifically, we investigated cerebellar resting state functional connectivity in Parkinson's patients ON and OFF dopaminergic medication compared to healthy controls of similar age and gender to evaluate if altered cerebellar connectivity is present at rest in Parkinson's disease. We tested if (1) there were differences in cerebellar-whole brain and cerebellar-cerebellar connectivity between Parkinson's patients ON medication, Parkinson's patients OFF medication, and matched controls, and (2) if the extent of an individual's cerebellar connectivity was related to their behavioral performance.

One prior study has examined cerebellar resting state functional connectivity in Parkinson's patients, but this study solely focused on connectivity from the dentate nucleus of the cerebellum and only tested Parkinson's patients OFF medication (Liu et al., 2013). Compared to controls, most dentate-cerebellar connectivity was enhanced in Parkinson's patients OFF medication. This reinforces what we found for other brain networks in Parkinson's disease using striatal seed regions (Kwak et al., 2010). Yet, assessment of motor dexterity and cognitive function were not evaluated, leaving the question open as to whether cerebellar connectivity changes are related to behavior.

Additionally, the current study examines connectivity differences using a more fine-grained, lobule-based analysis. We have recently applied this approach to the study of cerebellar connectivity in healthy young adults, revealing that disparate cerebellar lobules are associated with dissociable “cognitive” and “motor” networks (Bernard et al., 2012). In particular, anterior cerebellar lobules (i.e., Lobules I–IV and V) are associated with more “motor” cortical areas, whereas more posterior cerebellar lobules (i.e., Crus I, Crus II, and Lobules VIIb, IX, X) are associated with cortical regions that have more “cognitive” functions (see also Buckner et al., 2011; Buckner, 2013).

In sum, our approach will enable us to classify if differences in cerebellar resting state connectivity are present in Parkinson's patients, to examine if this resting cerebellar connectivity is modulated by medication state, and to determine if greater resting cerebellar connectivity is associated with fewer motor symptoms, improved cognition, and better motor performance. These results will inform our understanding of cerebellar alterations in Parkinson's disease.

## Materials and methods

### Participants

The same sample as reported in Kwak et al. (2010) was included in the present analysis, with one exception. One control participant had to be excluded due to inadequate imaging of the cerebellum. After this exclusion, there were 25 Parkinson's patients and 23 controls of similar age, sex distribution, and education. Parkinson's patients were screened by a neurologist to include those with mild-to-moderate stage Parkinson's disease, Hoehn and Yahr stages 1–2.5, based on the motor section of the Unified Parkinson's Disease Rating Scale (UPDRS; Hoehn and Yahr, 1967). The neurologist confirmed each patient's self-reported more-affected body side.

Parkinson's patients were scanned both ON and OFF medication in a counterbalanced manner. While ON medication, Parkinson's participants received 50 mg carbidopa and a combination of 200 mg L-DOPA and 50 mg carbidopa. While OFF medication, Parkinson's participants received 50 mg carbidopa and a placebo. All participants arrived in the OFF state, withdrawing from their medication 12–18 h prior to testing. The experimental sessions began 1 h after patients had taken either L-DOPA or the placebo. See Table 1 for characteristics of the sample. The University of Michigan Institutional Review Board approved this study; all participants provided written consent.

**Table 1:** **Demographic characteristics of the sample**.

**Group**	**n**	**Age (years)**	**Education (years)**	**% Male**	**UPDRS**		**MOCA**		**Grooved pegboard (ms)**			
					**ON**	**OFF**	**ON**	**OFF**	**More affected**		**Less affected**	
									**ON**	**OFF**	**ON**	**OFF**
Parkinson's	25	63.56 (7.83)	15.60 (2.69)	88%	17.44 (7.83)	18.64 (8.15)	26.08 (3.07)	26.08 (2.47)	116.96 (43.82)	128.08 (48.64)	114.28 (41.34)	116.16 (41.39)
Controls	23	64.00 (6.90)	15.77 (3.41)	83%	–	–	–	26.00 (2.37)	–	–	–	79.43 (14.17)

*Means reported with standard deviations in parentheses*.

## Materials

In addition to the resting state scans, participants also completed cognitive and motor assessments, including the motor section of the UPDRS (Hoehn and Yahr, 1967), the Montreal Cognitive Assessment (MOCA; Nasreddine et al., 2005), and the Grooved Pegboard task (Lafayette Instruments, Lafayette, IN, Model 32025). The UPDRS is a standardized measure of Parkinson's disease symptomatology, in which higher scores indicate worse disease severity. The MOCA is a set of neuropsychological assessments designed to detect mild cognitive impairment. Higher scores indicate better cognitive performance, and tasks include figure drawing, picture naming, verbal memory, verbal fluency, assessments of executive function, conceptual abstraction, and orientation to time and place. Finally, the Grooved Pegboard task is a timed measure of manual dexterity. Participants must correctly position and place pegs into their appropriate peg-holes as quickly as possible.

### Procedure

Participants completed both structural and functional MRI scans. During the 8-min functional resting state scan, participants viewed a black fixation cross on a white screen presented with a rear-projection visual display. Participants were asked to focus on the cross and not to think about anything in particular. Following the scan, participants completed the motor section of the UPDRS, the MOCA, and the Grooved Pegboard test.

#### fMRI data acquisition

Data were collected at the University of Michigan on a 3 T GE Signa MRI scanner. A total of 240 T2^*^-weighted BOLD images were acquired with a single-shot gradient-echo (GRE) reverse spiral pulse sequence (Glover and Law, 2001). The TR was set to 2 s and the TE was set to 30 ms. We collected 43 axial slices (flip angle = 90°) with a field of view (FOV) of 220 × 220 mm. This yielded a voxel size of 3.4 × 3.4 × 3 mm. The in-plane resolution was 3.4375 × 3.4375 mm, with a slice thickness of 3 mm. There was no gap factor. For the structural images, a 3D T1 axial overlay was acquired for anatomical localization. This overlay had a TR of 8.9 ms and a TE of 1.8 ms (flip angle = 15°, FOV = 220 × 220 mm, slice thickness = 3 mm, 43 slices; matrix = 256 × 256). The in-plane resolution was 0.86 × 0.86 mm, with no gap factor. To facilitate normalization, a 124-sliced (sagittal) inversion-prepped T1-weighted anatomical image was acquired using spoiled gradient-recalled (SPGR) acquisition in steady state imaging (flip angle = 15°, FOV = 256 × 256 mm, 1.2 mm slice thickness). The in-plane resolution was 1 × 1 mm, with no gap factor.

To monitor respiratory signal a pressure belt was placed around the participant's abdomen, and to measure cardiac signal, a pulse oximeter was placed on the participant's finger. The respiratory, cardiac, and fMRI data collection were synchronized.

#### fMRI data analysis

Images were preprocessed by the University of Michigan Functional MRI Laboratory. First, k-space outliers that were greater than two standard deviations from the mean were replaced with the average of their temporal neighbors. Second, the images were reconstructed with a field map correction to eliminate distortions from magnetic field inhomogeneity. Third, regression analyses were performed to remove physiological variations in the data due to cardiac and respiratory rhythms (Glover et al., 2000). We did not regress out global signal, which has been shown to be problematic (Murphy et al., 2009). Fourth, local sinc interpolation was used to correct slice timing differences (Oppenheim et al., 1999). Fifth, motion correction, using the 10th image as the reference, was performed with MCFLIRT in the fMRIB Software Library (Jenkinson et al., 2002). Head motion was less than 3 mm in the x-, y-, and z-directions for all participants, and, importantly, there was no difference in head motion between groups.

To average data across every patient's more affected body side, we flipped data for seven Parkinson's patients in the x-direction, so that the left side of the image is contralateral to the more affected body side. We flipped the data of six randomly selected control participants to ensure that this flipping did not account for any observed group differences. Next, the structural and functional images were skull-stripped using FSL and coregistered to a template brain using SPM8 for MATLAB (Version R2011A). These images were then normalized to MNI space using Advanced Normalization Tools (ANTS; Avants et al., 2008; Penn Image Computing and Science Lab, http://picsl.upenn.edu/software/ants/). The transformation was first applied to the structural SPGR images, then the resulting warp vectors were applied to the functional images. As in Bernard et al. (2013), the cerebellum was normalized separately from the whole brain to the spatially unbiased infra-tentorial (SUIT) template (Diedrichsen, 2006; Diedrichsen et al., 2009) using ANTS. Again, the structural images were normalized first, and the same warp vectors were applied to the functional cerebellar images. All data were smoothed using a 4 mm FWHM Gaussian kernel, after normalization. We intentionally applied a modest smoothing kernel because we wanted to avoid blurring the signal between neighboring cerebellar lobules, which are small structures.

Next, functional images were filtered using a dual-pass band-pass filter to leave frequencies of 0.01–0.16 Hz. Using masks created by the cerebellar SUIT atlas (Diedrichsen et al., 2009), we extracted the average time course of activity for each of the seed lobules of the cerebellum. To make the data analysis and interpretation more manageable we restricted our analyses to seven seed regions that included lobules shown to have involvement in motor and cognitive function. These seed regions were all in the right cerebellar hemisphere, including: Crus I, Crus II, and Lobules I–IV, V, VI, VIIIa, VIIIb. The SUIT atlas combines Lobules I, II, III, and IV, hence we refer to them as Lobules I–IV. The time course of each lobule was unit normalized to eliminate differences in means and variances between lobules.

Then, we created correlation maps between the average time course of activity in the seed lobule with the time courses of activity in every other voxel in the brain. Separate maps were created for the whole brain and for the cerebellum. Using Fisher's r-to-z transformation, we converted the functional connectivity correlation maps to z-scores. Finally, we used the functional connectivity z-scores from each participant to conduct within-group and between-group analyses in SPM8. These analyses revealed voxels exhibiting significant cerebellar functional connectivity (within-groups) or significant differences in the magnitude of cerebellar functional connectivity (between-groups) for each seed region. Behavioral regressions relating the strength of connectivity to performance outside the scanner were also conducted.

#### Statistical comparisons

First, we performed whole brain analyses, investigating voxels that showed significant connectivity with each seed region in the cerebellum. One-sample *t*-tests comparing connectivity to zero were conducted separately for the three groups: Parkinson's ON, Parkinson's OFF, and controls. Next, paired samples *t*-tests were run comparing cerebellar-whole brain connectivity between Parkinson's patients ON and OFF medication. Independent samples *t*-tests were then used to assess differences in cerebellar-whole brain connectivity between controls and the ON condition as well as between controls and the OFF condition. For within-group analyses, we used a family-wise error correction of 0.05 and an extent threshold of 100 voxels. For between-group analyses, we used an uncorrected *p*-value of 0.001 and an extent threshold of 50 voxels. We implemented a stricter threshold for within-group analyses to preempt indistinguishably large clusters. Also, because we ran separate cerebellar-cerebellar connectivity analyses using the SUIT template, for the whole brain analyses, we do not report connectivity within the cerebellum itself.

Behavioral regressions were then performed to relate cerebellar-whole brain connectivity to behavior. We used results from the between-subjects and within-subjects connectivity analyses as explicit inclusive masks to restrict our analysis to voxels that showed significant differences in connectivity (between-groups) or significant levels of connectivity (within-groups). We used SPM8 to run behavioral regressions, associating individual levels of connectivity with individual performance on the motor UPDRS, MOCA, and Grooved Pegboard tasks. Each task was entered in an independent analysis. An uncorrected alpha level of *p* < 0.001 and an extent threshold of 10 voxels was used for these behavioral regressions.

Next, within-cerebellar functional connectivity analyses were performed to investigate significant cerebellar-cerebellar connectivity at rest. The same between- and within-group analyses and behavioral regressions were run as reported above, but now we solely examined cerebellar-cerebellar connectivity using the cerebellar data that had been normalized to the SUIT atlas. The statistical alpha values and voxel extent thresholds were identical to those used for the whole brain analyses, with one exception: We used a stricter family-wise error rate of *p* < 0.001 for the cerebellar-cerebellar within-group analyses, along with an extent threshold of 100 voxels.

## Results

### Behavioral results

#### ON vs. OFF

Generally, Parkinson's patients had better behavioral performance when ON medication than when OFF medication. This behavioral difference was significant for the Grooved Pegboard completion time with the more affected hand, such that Parkinson's patients took longer when OFF medication, [*t*_(24)_ = 2.88, *p* = 0.008, *d* = 0.24]. However, differences in motor symptomatology when ON and OFF medication, as assessed by the UPDRS, did not reach significance, [*t*_(24)_ = 1.58, *p* = 0.127, *d* = 0.15]. Moreover, there were no significant differences between Parkinson's patients ON and OFF medication on the MOCA, [*t*_(24)_ = 0, *p* = 1, *d* = 0, or on the Grooved Pegboard task with the less affected hand, *t*_(24)_ = 0.41, *p* = 0.684, *d* = 0.05].

#### Parkinson's vs. controls

Compared to control participants, Parkinson's patients both ON and OFF medication were slower to complete the Grooved Pegboard. This was true when comparing control participants' dominant hand performance to both patients' more and less affected hand performance, all *p's* < 0.001. Also, there were no significant differences in MOCA scores for Parkinson's patients ON and OFF medication or controls, *p's* > 0.90, indicating that our sample of mild-to-moderate Parkinson's patients had similar cognitive functioning as their age- and sex-matched counterparts.

### Cerebellar-whole brain connectivity

#### Parkinson's ON medication

**Figure 1:**
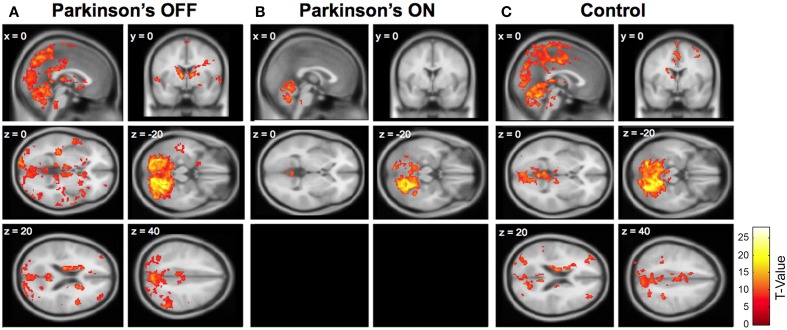
**Cerebellar connectivity between right Lobule VI (seed) and the whole brain in (A) Parkinson's patients OFF medication; (B) Parkinson's patients ON medication; and (C) older adult control participants**. Significant connectivity compared to zero is shown, using a family-wise error (FWE) correction of 0.05 and an extent threshold of 100 voxels. One sagittal section and one coronal section are shown for each group, followed by four horizontal sections. Two horizontal sections are omitted from the Parkinson's ON group because no significant cerebellar connectivity was present in these sections. Similar patterns of connectivity were found using other seed lobules (see Supplementary Tables 1, 2).

Limited significant cerebellar-whole brain connectivity was present in Parkinson's patients ON medication. See Supplementary Table 1 and Figure 1. Of the seven seed lobules analyzed, only Lobules I–IV and Lobule V exhibited significant levels of functional connectivity. Lobules I–IV had the greatest functional connectivity with the thalamus, whereas Lobule V had the greatest functional connectivity with the posterior cingulate.

#### Parkinson's OFF medication

In sharp contrast, Parkinson's patients OFF medication had widespread cerebellar functional connectivity. See Supplementary Table 2 and Figure 1. All of the cerebellar seed regions except right Lobule VIIIb showed significant functional connectivity with numerous cortical and subcortical areas. For instance, right Crus I had significant connectivity with the precuneus, caudate, superior frontal gyrus, middle temporal gyrus, and middle frontal gyrus. Moreover, there was significant functional connectivity between Lobule V and the inferior parietal gyrus, rostral cingulate, middle temporal gyrus, middle frontal gyrus, and postcentral gyrus. See Supplementary Table 2 for a complete list. This widespread connectivity provides some support for an anterior-posterior divide (e.g., Stoodley and Schmahmann, 2009; Stoodley et al., 2012; Bernard et al., 2014b), such that anterior lobules exhibited connectivity with motor areas and posterior lobules exhibited connectivity with cognitive areas. However, the extensive connectivity between each seed region and the rest of the brain also included considerable connectivity beyond these motor and cognitive distinctions.

#### Controls

Older adult controls showed intermediate levels of cerebellar connectivity. They exhibited qualitatively greater cerebellar connectivity than Parkinson's patients ON medication and less cerebellar connectivity compared to Parkinson's patients OFF medication. Because the older adult connectivity results are similar to those reported in Bernard et al. (2013) we do not include a redundant table (but see Figure 1).

#### Parkinson's ON vs. OFF

Paired-samples *t*-tests revealed several regions of greater cerebellar connectivity when Parkinson's patients were OFF as opposed to ON medication. These differences were only observed when using the right Lobule VI seed, which displayed greater functional connectivity to the cuneus, precuneus, and supramarginal gyrus in Parkinson's patients OFF than ON medication. See Table 2. There were no significant clusters for the ON > OFF contrast. These results indicate that Parkinson's patients OFF medication had greater cerebellar resting state functional connectivity than the same patients ON medication.

**Table 2:** **MNI coordinates of the local maxima of brain regions showing significantly greater cerebellar connectivity in Parkinson's patients OFF medication than ON medication**.

**Seed**	**Region**	**BA**	**MNI coordinates**			**Cluster size**	***T*-Value**	**Contrast**	**Interpretation**
			**x**	**y**	**z**				
Right VI	**Cuneus**	**18**	**0**	**−92**	**20**	**73**	**6.22**	OFF > ON	Hyperconnectivity OFF vs. ON
	Calcarine Gyrus	18	−2	−92	12	73	4.33	OFF > ON	Hyperconnectivity OFF vs. ON
	**Precuneus**	**5**	**−12**	**−58**	**66**	**105**	**5.42**	OFF > ON	Hyperconnectivity OFF vs. ON
	Precuneus	7	−8	−66	68	105	4.68	OFF > ON	Hyperconnectivity OFF vs. ON
	**Supramarginal Gyrus**	**40**	**64**	**−34**	**46**	**58**	**5.04**	OFF > ON	Hyperconnectivity OFF vs. ON
Right VI	**Vermis VI**	**–**	**0**	**−77**	**−20**	**517**	**5.85**	OFF > ON	Hyperconnectivity OFF vs. ON
	Vermis VI	**–**	−2	−71	−26	517	4.54	OFF > ON	Hyperconnectivity OFF vs. ON
	**Left Crus I**	**–**	**−20**	**−82**	**−29**	**215**	**5.05**	OFF > ON	Hyperconnectivity OFF vs. ON

*Peak values for each cluster are shown in bold. Cerebellar-whole brain connectivity is reported first. Within-cerebellar connectivity is reported second*.

#### Parkinson's ON vs. controls

Likewise, Parkinson's patients ON medication had significantly less cerebellar-whole brain connectivity than controls. No regions exhibited greater connectivity in the ON group. Instead, four cerebellar seed regions showed greater connectivity in controls. For instance, in controls there was significantly more connectivity between right Crus I and the middle frontal gyrus, superior frontal gyrus, and angular gyrus, and there was significantly more functional connectivity between right Lobule VI and the middle temporal gyrus, postcentral gyrus, and the supramarginal gyrus. See Table 3 for a complete list. The significant functional connectivity between Crus I and the frontal gyri is consistent with the previously observed involvement of Crus I in cognitive processing (Stoodley and Schmahmann, 2009; Bernard et al., 2012; Stoodley et al., 2012). Overall, these results indicate decreased cerebellar connectivity in Parkinson's patients ON L-DOPA medication.

**Table 3:** **MNI coordinates of the local maxima of regions showing significant differences in cerebellar functional connectivity between Parkinson's patients ON medication and older adult (OA) controls**.

**Seed**	**Region**	**BA**	**MNI coordinates**			**Cluster size**	***T*-Value**	**Contrast**	**Interpretation**
			**x**	**y**	**z**				
Right Crus I	**Middle Frontal Gyrus**	**6**	**28**	**10**	**48**	**60**	**5.20**	OA > ON	Hypoconnectivity in ON vs. OA
	**Superior Frontal Gyrus**	**6**	**−22**	**4**	**58**	**93**	**4.51**	OA > ON	Hypoconnectivity in ON vs. OA
	Superior Frontal Gyrus	6	−24	−4	64	93	4.43	OA > ON	Hypoconnectivity in ON vs. OA
	**Angular Gyrus**	**39**	**−40**	**−66**	**28**	**65**	**4.37**	OA > ON	Hypoconnectivity in ON vs. OA
	Angular Gyrus	39	−46	−58	26	65	3.95	OA > ON	Hypoconnectivity in ON vs. OA
	Angular Gyrus	39	−54	−62	28	65	3.90	OA > ON	Hypoconnectivity in ON vs. OA
Right Crus II	**Lingual Gyrus**	**17**	**−4**	**−64**	**6**	**106**	**4.86**	OA > ON	Hypoconnectivity in ON vs. OA
	Lingual Gyrus	17	8	−64	6	106	4.66	OA > ON	Hypoconnectivity in ON vs. OA
	Lingual Gyrus	18	−4	−72	−2	106	4.13	OA > ON	Hypoconnectivity in ON vs. OA
	Precentral Gyrus	**6**	**−16**	**−8**	**68**	**93**	**4.71**	OA > ON	Hypoconnectivity in ON vs. OA
	Superior Frontal Gyrus	6	−20	4	60	93	4.00	OA > ON	Hypoconnectivity in ON vs. OA
	**Calcarine Gyrus**	**30**	**12**	**−56**	**14**	**62**	**4.19**	OA > ON	Hypoconnectivity in ON vs. OA
	Precuneus	23	10	−50	24	62	4.13	OA > ON	Hypoconnectivity in ON vs. OA
	**Paracentral Lobule**	**4**	**−8**	**−28**	**76**	**63**	**4.12**	OA > ON	Hypoconnectivity in ON vs. OA
	Paracentral Lobule	4	−2	−26	70	63	3.97	OA > ON	Hypoconnectivity in ON vs. OA
	Paracentral Lobule	4	−16	−22	72	63	3.83	OA > ON	Hypoconnectivity in ON vs. OA
	Middle Frontal Gyrus	**6**	**26**	**8**	**44**	**69**	**4.11**	OA > ON	Hypoconnectivity in ON vs. OA
	Middle Frontal Gyrus	6	30	0	46	69	3.64	OA > ON	Hypoconnectivity in ON vs. OA
Right VI	**Middle Temporal Gyrus**	**37**	**−50**	**−56**	**8**	**60**	**5.42**	OA > ON	Hypoconnectivity in ON vs. OA
	Inferior Temporal Gyrus	37	−50	−52	−4	60	3.32	OA > ON	Hypoconnectivity in ON vs. OA
	**Postcentral Gyrus**	**43**	**−64**	**−2**	**22**	**81**	**4.33**	OA > ON	Hypoconnectivity in ON vs. OA
	**Supramarginal Gyrus**	**48**	**−54**	**−40**	**28**	**68**	**3.99**	OA > ON	Hypoconnectivity in ON vs. OA
	Supramarginal Gyrus	40	−62	−34	38	68	3.95	OA > ON	Hypoconnectivity in ON vs. OA
	Superior Temporal Gyrus	48	−64	−42	24	68	3.75	OA > ON	Hypoconnectivity in ON vs. OA
Right VIIIa	**Middle Temporal Gyrus**	**37**	**−48**	**−56**	**10**	**125**	**5.00**	OA > ON	Hypoconnectivity in ON vs. OA
	Middle Temporal Gyrus	37	−56	−60	14	125	4.94	OA > ON	Hypoconnectivity in ON vs. OA
	**Calcarine Gyrus**	**17**	**10**	**−80**	**16**	**71**	**4.68**	OA > ON	Hypoconnectivity in ON vs. OA
	Calcarine Gyrus	17	18	−82	12	71	3.45	OA > ON	Hypoconnectivity in ON vs. OA
Right Crus I	**Right Crus II**	**−**	**31**	**−77**	**−48**	**71**	**3.80**	OA > ON	Hypoconnectivity ON vs. OA
Right I–IV	**Left I–IV**	**−**	**−10**	**−35**	**−21**	**53**	**4.08**	ON > OA	Greater connectivity ON vs. OA
Right V	**Left Crus II**	**−**	**−7**	**−76**	**−34**	**97**	**4.44**	ON > OA	Greater connectivity ON vs. OA
Right VI	**Right Crus II**	**−**	**30**	**−78**	**−47**	**125**	**4.54**	OA > ON	Hypoconnectivity ON vs. OA
	**Left Crus II**	**−**	**−32**	**−79**	**−45**	**130**	**4.17**	OA > ON	Hypoconnectivity ON vs. OA
	**Right VIIb**	**−**	**43**	**−41**	**−48**	**118**	**4.10**	OA > ON	Hypoconnectivity ON vs. OA
	**Vermis VIIIa**	**−**	**−2**	**−72**	**−45**	**54**	**3.87**	OA > ON	Hypoconnectivity ON vs. OA
Right VIIIb	**Vermis VI**	**−**	**−2**	**−74**	**−27**	**55**	**3.90**	OA > ON	Hypoconnectivity ON vs. OA

*Peak values for each cluster are shown in bold. Cerebellar-whole brain connectivity is reported first. Within-cerebellar connectivity is reported second*.

#### Parkinson's OFF vs. controls

Finally, between-group comparisons of Parkinson's patients OFF medication and controls revealed greater connectivity in the OFF group. Specifically, right Lobule V exhibited significantly more functional connectivity with the inferior temporal gyrus [Brodmann's Area (BA) 20, MNI (54, −16, −34), cluster size = 196 voxels, *t* = 5.50; BA 20, MNI (54, −34, −28), cluster size = 196 voxels, *t* = 4.12]. No other significant local maxima were present for the OFF > Control contrast, and zero significant clusters were present for the Control > OFF contrast.

### Whole brain behavioral regressions

#### Between-group analyses

No regions with 10 or more voxels showed significant relationships to behavior, when restricting the voxel space to those that exhibited significant between-group differences in cerebellar-whole brain connectivity.

#### Parkinson's ON medication

Since Parkinson's patients ON medication did not show much significant cerebellar connectivity outside the cerebellum, expectedly, no significant behavioral regressions emerged for this ON analysis.

#### Parkinson's OFF medication

Two clusters showed significant relationships between connectivity and behavioral performance for Parkinson's patients OFF medication. Specifically, increased functional connectivity between right Crus I and the lingual gyrus was associated with faster Grooved Pegboard performance using the more affected hand. This may reflect the need for cognitive-visual processing to perform the task. However, increased functional connectivity between right Lobule V and the precuneus was associated with worse MOCA performance. See Table 4. Thus, in Parkinson's patients OFF medication greater cerebellar connectivity was both beneficial and detrimental.

**Table 4:** **MNI coordinates for areas showing significant relationships between cerebellar functional connectivity and behavioral performance in Parkinson's patients ON and OFF medication**.

**Group**	**Seed**	**Region**	**BA**	**Task**	**MNI coordinates**			**Cluster size**	**T**	**±**	**Interpretation**
					**x**	**y**	**z**				
OFF	Right Crus I	**Lingual Gyrus**	**17**	Peg More Aff OFF	**6**	**−80**	**−6**	**12**	**4.96**	Neg	Higher connectivity, faster pegboard.
	Right V	**Precuneus**	**7**	MOCA OFF	**4**	**−66**	**58**	**29**	**5.93**	Neg	Higher connectivity, greater cognitive impairment.
ON	Right Crus II	**Right VI**	–	MOCA ON	**30**	**−64**	**−24**	**36**	**4.37**	Pos	Higher connectivity, better cognition.
	Right V	**Right VI**	–	Peg More Aff ON	**20**	**−57**	**−14**	**90**	**4.97**	Neg	Higher connectivity, faster pegboard.
		**Left V**	–	Peg Less Aff ON	**−15**	**−49**	**−18**	**20**	**3.81**	Neg	Higher connectivity, faster pegboard.
	Right VI	**Right V**	–	UPDRS ON	**23**	**−44**	**−20**	**26**	**4.25**	Neg	Higher connectivity, less disease severity.
		**Left V**	–	Peg Less Aff ON	**−14**	**−49**	**−17**	**40**	**5.12**	Neg	Higher connectivity, faster pegboard.
	Right VIIIa	**Right VIIIb**	–	MOCA ON	**28**	**−42**	**−45**	**10**	**3.93**	Pos	Higher connectivity, better cognition.
OFF	Right Crus I	**Vermis VIIIb**	–	Peg More Aff OFF	**6**	**−62**	**−36**	**10**	**4.02**	Neg	Higher connectivity, faster pegboard.
		**Left Crus I**	–	Peg More Aff OFF	**−40**	**−58**	**−28**	**13**	**3.83**	Neg	Higher connectivity, faster pegboard.
	Right Crus II	**Right Crus I**	–	UPDRS OFF	**33**	**−66**	**−33**	**56**	**4.43**	Pos	Higher connectivity, worse disease severity.
		**Right Crus I**	–	Peg Less Aff OFF	**35**	**−65**	**−30**	**10**	**3.72**	Pos	Higher connectivity, slower pegboard.
	Right I–IV	**Left I–IV**	–	Peg More Aff OFF	**−11**	**−34**	**−22**	**482**	**7.37**	Neg	Higher connectivity, faster pegboard.
		Left I–IV	–	Peg More Aff OFF	−9	−42	−17	482	5.28	Neg	Higher connectivity, faster pegboard.
		Left V	–	Peg More Aff OFF	−13	−58	−14	482	4.42	Neg	Higher connectivity, faster pegboard.
		**Left I–IV**	–	Peg Less Aff OFF	**−9**	**−41**	**−16**	**37**	**4.26**	Neg	Higher connectivity, faster pegboard.
	Right V	**Vermis VI**	–	UPDRS OFF	**6**	**−71**	**−19**	**12**	**3.66**	Neg	Higher connectivity, less disease severity.
		**Left I–IV**	–	Peg More Aff OFF	**−7**	**−45**	**−15**	**37**	**4.25**	Neg	Higher connectivity, faster pegboard.
		**Left V**	–	Peg More Aff OFF	**−14**	**−57**	**−15**	**11**	**3.94**	Neg	Higher connectivity, faster pegboard.
		**Left V**	–	Peg Less Aff OFF	**−16**	**−52**	**−20**	**11**	**3.68**	Neg	Higher connectivity, faster pegboard.
	Right VI	**Left Crus I**	–	UPDRS OFF	**−35**	**−72**	**−40**	**35**	**4.90**	Neg	Higher connectivity, less disease severity.
		**Left Crus I**	–	Peg Less Aff OFF	**−37**	**−66**	**−37**	**15**	**3.93**	Neg	Higher connectivity, faster pegboard.
		**Left V**	–	Peg Less Aff OFF	**−23**	**−40**	**−29**	**18**	**3.74**	Pos	Higher connectivity, slower pegboard.
	Right VIIIa	**Right VIIb**	–	MOCA OFF	**37**	**−52**	**−48**	**21**	**4.36**	Pos	Higher connectivity, better cognition.

*Peak values for each cluster are shown in bold. Cerebellar-whole brain connectivity is reported first. Within-cerebellar connectivity is reported second. The abbreviations “Peg” and “Aff” connote “Grooved Pegboard performance” and “affected hand,” respectively*.

#### Controls

Controls showed one instance where increased resting state cerebellar connectivity was associated with better performance: Greater connectivity between right Lobule VI and the precuneus [BA 7; MNI = (−6, −60, 46), cluster size = 14; *t* = 5.18] was associated with faster Grooved Pegboard completion with the dominant hand. Although we should be cautious in the interpretation of these results due to the fact that the relationship was only found in one cluster of voxels, these results suggest that greater cerebellar connectivity may assist motor performance in healthy older individuals.

### Cerebellar-cerebellar connectivity

#### Within-group analyses

Our within-group cerebellar-cerebellar connectivity analyses documented extensive within-cerebellar connectivity for all groups. Tables of these within-group analyses are included in Supplementary Tables 3, 4.

#### Parkinson's ON vs. OFF

As in the whole brain analyses, the within-cerebellar analyses revealed that Parkinson's patients ON medication exhibited less within-cerebellar functional connectivity than Parkinson's patients OFF medication. There were no significant clusters present for the ON > OFF contrast. Instead, the opposite contrast revealed significantly greater connectivity between right Lobule VI and both the vermis near Lobule VI and left Crus I. See Table 2. Compared to when OFF medication, Parkinson's patients ON medication had reduced cerebellar-cerebellar functional connectivity.

#### Parkinson's ON vs. controls

Independent samples *t*-tests revealed decreased cerebellar-cerebellar functional connectivity in Parkinson's patients ON medication compared to controls. See Table 3. Specifically, Parkinson's patients ON medication had less connectivity between right Crus I and right Crus II, between right Lobule VIIIb and the vermis near Lobule VI, and between right Lobule VI and right Crus II, left Crus II, right Lobule VIIb, and the vermis near Lobule VIIIa. Nevertheless, there was also some evidence for increased cerebellar-cerebellar connectivity in Parkinson's patients ON medication compared to controls. Parkinson's patients ON medication had greater functional connectivity between right Lobule I–IV and left Lobule I–IV and between right Lobule V and left Crus II. Thus, while the pattern of decreased cerebellar-cerebellar connectivity is most prevalent in Parkinson's patients ON medication, we also note instances of increased cerebellar-cerebellar connectivity.

#### Parkinson's OFF vs. controls

Like the whole-brain connectivity analysis, the within-cerebellar analysis revealed evidence for greater functional connectivity in Parkinson's patients OFF medication than controls. For example, right Lobule V exhibited greater functional connectivity with left Lobule VI, left Crus I, and right Crus I in Parkinson's patients OFF medication compared to controls. Nonetheless, of the 10 significant clusters, we note two instances when older adults showed greater within-cerebellar connectivity than Parkinson's patients OFF medication. See Table 5.

**Table 5:** **MNI coordinates of regions showing significant differences in cerebellar-cerebellar connectivity between Parkinson's patients OFF medication and older adult (OA) controls**.

**Seed**	**Region**	**MNI coordinates**			**Cluster size**	***T*-Value**	**Contrast**	**Interpretation**
		**x**	**y**	**z**				
Right Crus I	**Vermis IX**	**5**	**−53**	**−32**	**52**	**3.86**	OFF > OA	Hyperconnectivity OFF vs. OA
Right I–IV	**Right VIIIb**	**21**	**−50**	**−49**	**109**	**4.36**	OA > OFF	Greater connectivity in OA than OFF
	**Left Crus I**	**−17**	**−75**	**−32**	**84**	**4.01**	OFF > OA	Hyperconnectivity OFF vs. OA
Right V	**Left VI**	**−33**	**−54**	**−32**	**61**	**3.99**	OFF > OA	Hyperconnectivity OFF vs. OA
	**Left Crus I**	**−16**	**−76**	**−34**	**63**	**3.95**	OFF > OA	Hyperconnectivity OFF vs. OA
	**Right Crus I**	**16**	**−82**	**−24**	**64**	**3.80**	OFF > OA	Hyperconnectivity OFF vs. OA
Right VI	**Right IX**	**7**	**−48**	**−34**	**50**	**4.06**	OFF > OA	Hyperconnectivity OFF vs. OA
Right VIIIb	**Left VIIb**	**−35**	**−55**	**−54**	**184**	**4.62**	OFF > OA	Hyperconnectivity OFF vs. OA
	**Right VI**	**9**	**−63**	**−24**	**52**	**4.17**	OFF > OA	Hyperconnectivity OFF vs. OA
	**Right I–IV**	**26**	**−31**	**−30**	**180**	**4.79**	OA > OFF	Greater connectivity in OA than OFF

*Peak values for each cluster are shown in bold*.

### Within-cerebellum behavioral regressions

#### Between-group analyses

No clusters with ten or more voxels showed significant relationships to behavior when restricting the voxel space to those that exhibited significant between-group differences in cerebellar-cerebellar connectivity.

#### Parkinson's ON medication

Data from Parkinson's patients ON medication consistently revealed that greater cerebellar-cerebellar connectivity was associated with improved behavioral performance. Greater levels of connectivity were associated with better cognitive performance on the MOCA, faster Grooved Pegboard completion, as well as reduced disease severity, measured by the motor section of the UPDRS. For example, individuals with greater connectivity between right Crus II and right Lobule VI had better MOCA scores. Additionally, greater connectivity between right Lobule V and right Lobule VI was associated with faster Grooved Pegboard completion with the more affected hand. Moreover, greater right Lobule VI-right Lobule V connectivity was related to reduced UPDRS motor symptomatology. See Table 4 for a complete list. Although Parkinson's patients ON medication showed decreased connectivity compared to controls and compared to when they were OFF medication, when ON medication, those participants who had higher levels of cerebellar-cerebellar connectivity consistently exhibited better behavioral performance.

#### Parkinson's OFF medication

Similarly, for Parkinson's patients OFF medication, we found strong evidence that higher levels of cerebellar-cerebellar connectivity were associated with better behavioral performance. Of the 14 clusters that showed significant relationships to behavior, 11 clusters provided support for a compensatory hypothesis. For instance, greater connectivity between right Lobules I–IV and left Lobules I–IV predicted faster Grooved Pegboard completion with the more affected hand. Moreover, people with greater resting state connectivity between right Lobule VI and left Crus I exhibited fewer Parkinson's symptoms, as well as faster Pegboard completion times. Nevertheless, greater connectivity between right Crus II and right Crus I was associated with worse disease severity, for example. See Table 4. Therefore, our data demonstrate considerable evidence for compensatory within-cerebellar connectivity, but we also acknowledge that this compensatory finding was not universal. Albeit fewer, there were some instances where greater within-cerebellar connectivity was detrimental.

#### Controls

One cluster showed a significant relationship to behavior for control participants. Those with greater connectivity between right Crus I and right Crus II displayed faster Grooved Pegboard completion with their dominant hand [MNI coordinates: (8, −90, −28); cluster size = 12, *t* = 4.11]. In control participants, although relationships to behavior were much less prominent, the one relationship that emerged suggested that greater resting cerebellar-cerebellar connectivity was beneficial.

## Discussion

The present project compared lobule-based cerebellar resting state functional connectivity in Parkinson's patients ON and OFF medication and healthy controls, and related levels of cerebellar connectivity to cognitive and motor performance. Parkinson's patients OFF medication displayed greater levels of cerebellar-whole brain and cerebellar-cerebellar connectivity, whereas Parkinson's patients ON medication displayed decreased levels of cerebellar-whole brain and cerebellar-cerebellar connectivity, compared to controls. Furthermore, greater cerebellar connectivity was most frequently associated with improved behavior, especially for cerebellar-cerebellar connectivity, although several detrimental relationships between connectivity and behavior were observed.

### Cerebellar connectivity spectrum

When comparing cerebellar-whole brain connectivity between groups, Parkinson's patients OFF medication consistently showed greater cerebellar connectivity than Parkinson's patients ON medication and controls. Likewise, when comparing within-cerebellar connectivity, higher levels of functional connectivity were again found in the OFF group.

Similarly, limited cerebellar connectivity was observed in Parkinson's patients ON medication. When compared to both controls and Parkinson's patients OFF medication, they had significantly less cerebellar connectivity. This finding was universal when comparing cerebellar-whole brain connectivity. However, for cerebellar-cerebellar connectivity, we note that although Parkinson's patients ON medication had reduced connectivity compared to Parkinson's patients OFF medication in all instances, the control comparison revealed two (of eight) occasions when the opposite pattern was observed. In the majority of instances, however, Parkinson's patients ON medication had reduced levels of cerebellar connectivity.

Thus, the following spectrum of cerebellar connectivity emerged: Parkinson's patients OFF medication displayed the most widespread cerebellar connectivity; older adult controls displayed a middle level of cerebellar connectivity; and Parkinson's patients ON medication displayed reduced cerebellar connectivity. This spectrum that varies with medication state is largely consistent with prior studies that used different seed regions (Kwak et al., 2010; Palmer et al., 2010; Hacker et al., 2012; Liu et al., 2013). Although the alterations in cerebellar connectivity are most pronounced in the PD ON group, such that they exhibit decreased cerebellar connectivity, the fact that we observed statistically significant increases in connectivity in Parkinson's patients OFF medication is noteworthy, considering that other clinical patient groups (e.g., schizophrenia, autism spectrum disorders) have exhibited decreased connectivity (Monk et al., 2009; Vercammen et al., 2010; Bernard et al., 2014a).

### Relationship to behavior

We also investigated whether cerebellar connectivity levels were related to individual variations in behavioral performance. Interestingly, increased levels of cerebellar connectivity were most commonly related to improved performance, particularly for within-cerebellar connectivity, supporting the proposition of compensatory cerebellar involvement in Parkinson's disease (e.g., Wu et al., 2009; Palmer et al., 2010). Nevertheless, in several instances, greater cerebellar connectivity was associated with worse performance. Thus, cerebellar involvement in Parkinson's disease may also reflect detrimental pathophysiological changes of the cerebellum and/or striatum (e.g., Martinu and Monchi, 2013).

One interpretation of the mixed results is that, at a certain level, reliance on the cerebellum is no longer feasible to compensate for other dysfunctional neural systems (i.e., the striatum), and cerebellar recruitment becomes affiliated with impaired behavior. This interpretation has been discussed in models of healthy cognitive aging, regarding compensatory frontal recruitment (e.g., Cabeza et al., 2002; Reuter-Lorenz and Cappell, 2008; Reuter-Lorenz and Park, 2010). However, we note that models of aging are most commonly supported by analyses of neural *activation*, whereas our current data involves *functional connectivity* differences.

### Relationship to medication state

Based on the absolute functional connectivity levels and the relationships between connectivity and behavior, our data connote that L-DOPA medication may overcorrect for dopamine depletion. That is, although the L-DOPA medication decreased (or arguably corrected) the hyperconnectivity present when patients were OFF medication, instead of resulting in a more typical cerebellar connectivity pattern, the L-DOPA medication promoted weaker cerebellar connectivity than was observed in control participants.

Additional evidence for overcorrection of L-DOPA is apparent in the behavioral regression analyses. Frequently, individuals who exhibited higher cerebellar connectivity performed better on the cognitive assessment, Grooved Pegboard, and UPDRS, especially for within-cerebellar connectivity. Thus, although generally showing decreased connectivity, those Parkinson's patients ON medication who showed the greatest levels of cerebellar-cerebellar connectivity consistently had better cognition, reduced disease severity, and increased manual dexterity, suggesting that maintaining greater levels of cerebellar connectivity was beneficial.

Evidence of L-DOPA overcorrection is not previously unfounded. For instance, Lewis et al. (2007) report that L-DOPA medication overcorrected cerebellar hypoactivation in Parkinson's patients compared to their twin counterparts during an internally guided finger-tapping task. Kwak et al. (2010) similarly found that increased levels of striatal-cortical functional connectivity in Parkinson's patients OFF medication were overcorrected by L-DOPA.

These overcorrective outcomes relate to the mixed beneficial and detrimental effects of L-DOPA found in the literature. Although L-DOPA improves *motor* symptoms fairly consistently (e.g., Hornykiewicz, 1974), varying levels of basal dopamine, task demands, and individual differences contribute to the inconsistently beneficial and harmful effects of L-DOPA on *cognition* (see Cools, 2006). In accord with this disparity between motor and cognitive outcomes, we find that Grooved Pegboard completion times were faster when patients were ON medication. However, the weaker levels of cerebellar connectivity in Parkinson's patients ON medication and the significant relationship between weaker connectivity and poorer cognition, implicate some detrimental effects of L-DOPA on cognition.

### Cerebellar connectivity patterns

Prior work detailing general patterns of cerebellar connectivity has distinguished lobules that are primarily involved in motor and cognitive processing (Bernard et al., 2012). Interestingly, in the present analyses, connectivity between regions of the cerebellum that subserve similar functions was typically associated with better performance. For example, right Lobule I–IV was positively correlated with left Lobule I–IV and left Lobule V, and this was associated with faster Grooved Pegboard completion. Lobules I–IV have exhibited connectivity with cortical motor areas, as has Lobule V (Bernard et al., 2012), and these regions have been implicated in motor performance (Stoodley and Schmahmann, 2009; Stoodley et al., 2012; Bernard and Seidler, 2013). Similarly, in Parkinson's patients ON medication, connectivity between right Crus II and right Lobule VI was associated with better cognition. These regions have been implicated in cognitive function (Stoodley and Schmahmann, 2009; Stoodley et al., 2012), and are part of networks that include prefrontal and parietal components (Buckner et al., 2011; Bernard et al., 2012). From these patterns it seems that, in some cases, Parkinson's patients may rely on redundant networks in a compensatory manner.

Though we do not see this extensively at the cortical level, this may be due to statistical thresholding, and we suggest that within-cerebellar connectivity may be driven by cortical connections. Given the closed-loop circuitry of the structure (Kelly and Strick, 2003; Strick et al., 2009), intra-cerebellar functional connectivity is likely the result of similar cortical targets. Thus, differences in cerebellar-cerebellar connectivity seen in Parkinson's patients both ON and OFF medication relative to controls are likely due to broad alterations in the functional network targets of these regions. In essence, the altered cerebellar connectivity patterns we observed may be due to altered interactions between striatal-thalamo-cortical and cerebello-thalamo-cortical pathways in Parkinson's patients (cf. Kishore and Popa, 2014).

### Limitations and future directions

The present experiment provides novel and important findings relevant to the role of the cerebellum in Parkinson's disease. Nevertheless, we acknowledge several limitations. First, we only performed connectivity analyses in seven cerebellar lobules. Although we feel that this methodological decision made the report more concise and consequently the interpretation more manageable, future research could similarly assess cerebellar connectivity using the remaining cerebellar lobules as seeds. Given the general consistency of our findings, however, we would predict similar results when using different cerebellar seed regions.

Additionally, our study focused on individuals with mild to moderate stage Parkinson's disease. Correspondingly, we are unable to generalize our findings to individuals with more severe symptoms. We also note that we did not detect significant improvement in UPDRS motor performance when participants were ON medication. This could indicate that participants may not have been in their best-medicated state when given a controlled medication dose. Nevertheless, the fact that behavioral differences were observed on the Grooved Pegboard task and that functional differences were observed in the connectivity analyses suggests that the standardized dose of medication had an impact. Future research could assess whether administration of personalized medication leads to similar results.

Moreover, the current functional connectivity analyses were conducted at rest, rather than when completing a specific cognitive or motor task. Nonetheless, several pieces of evidence suggest that our resting state findings are applicable to different task states. First, we find that resting state cerebellar connectivity levels were significantly correlated with behavioral task performance. Moreover, prior research using task-based connectivity analyses found similar patterns of increased cerebellar connectivity in Parkinson's patients OFF medication (Palmer et al., 2010). Thus, our resting state analyses appear to be relevant to task-based states as well.

## Conclusions

The current project used lobule-based resting-state functional connectivity analyses to further evaluate the role of the cerebellum in Parkinson's disease. Parkinson's patients OFF medication displayed greater cerebellar-whole brain and cerebellar-cerebellar connectivity, and Parkinson's patients ON medication displayed reduced cerebellar-whole brain and cerebellar-cerebellar connectivity. This increased cerebellar connectivity in PD patients OFF medication is largely consistent with task-based cerebellar connectivity analyses (Palmer et al., 2010) and analyses using different cerebellar (Liu et al., 2013) and striatal seeds (Kwak et al., 2010). The decreased cerebellar connectivity in PD patients ON medication is consistent with analyses using striatal seeds (Kwak et al., 2010; Hacker et al., 2012). Moreover, we evaluated relationships between the degree of cerebellar connectivity and behavioral performance and found that greater cerebellar connectivity was most often associated with improved behavior, particularly for within-cerebellar connectivity. This relationship between increased cerebellar connectivity and enhanced cognitive and motor performance favors a compensatory view. However, in several instances, greater cerebellar connectivity was associated with poorer behavior. Overall, the present study demonstrates medication-variant cerebellar functional connectivity in Parkinson's patients at rest, further characterizing the altered cerebellar function in Parkinson's disease.

## Author contributions

RS, YK, SF, and JB designed this work. YK, NB, MM, and PD completed data acquisition and assessment of patients. SF analyzed the data, with the assistance of JB, SP, YK, and RS. SF wrote the manuscript. All authors critically edited the manuscript and approved the final version.

### Conflict of interest statement

The authors declare that the research was conducted in the absence of any commercial or financial relationships that could be construed as a potential conflict of interest.
