# Supplementary material for: Altered cerebellar connectivity in Parkinson's patients ON and OFF L-DOPA medication
Source: Front Hum Neurosci. 2015 Apr 21;9:214. doi: 10.3389/fnhum.2015.00214 (PMC4405615; doi:10.3389/fnhum.2015.00214)
Supplement: Supplementary file 1 [file Table1.DOCX]

***Supplementary Material***

**Altered cerebellar connectivity in Parkinson’s patients ON and OFF L-DOPA medication**

**Sara B. Festini^1,2^*, Jessica A. Bernard^3^, Youngbin Kwak^4^, Scott Peltier^5^, Nicolaas I. Bohnen^6,7,8^, Martijn L. T. M. Müller^6^, Praveen Dayalu^7^, & Rachael D. Seidler^9,2^***

^1^Center for Vital Longevity, School of Behavioral and Brain Sciences, University of Texas at Dallas, Dallas, TX, USA

^2^Department of Psychology, University of Michigan, Ann Arbor, MI, USA

^3^Department of Psychology & Neuroscience, University of Colorado Boulder, Boulder, CO, USA

^4^Department of Psychological and Brain Sciences, University of Massachusetts Amherst, Amherst, MA, USA

^5^Functional MRI Laboratory, Department of Biomedical Engineering, University of Michigan, Ann Arbor, MI, USA

^6^Department of Radiology, University of Michigan, Ann Arbor, MI, USA

^7^Department of Neurology, University of Michigan, Ann Arbor, MI, USA

^8^Geriatric Research, Education and Clinical Center (GRECC), VA Ann Arbor, Ann Arbor, MI, USA

^9^School of Kinesiology, University of Michigan, Ann Arbor, MI, USA

*** Correspondence:** Sara B. Festini, Center for Vital Longevity, School of Behavioral and Brain Sciences, University of Texas at Dallas, 1600 Viceroy Drive Suite 800, Dallas, TX, 75235, USA. sara.festini@utdallas.edu

*** Correspondence:** Rachael D. Seidler, School of Kinesiology, University of Michigan, 401 Washtenaw Ave., Ann Arbor, MI, 48109-1043, USA.

rseidler@umich.edu

1. **Supplementary Tables**

**Supplementary Table 1.** MNI coordinates of the local maxima of brain regions showing significant functional connectivity with cerebellar seed regions in Parkinson’s patients ON medication. Peak values for each cluster are shown in bold.

| **Seed** | **Region** | **BA** | **MNI coordinates** | | | **Cluster size** | **T-Value** |
| --- | --- | --- | --- | --- | --- | --- | --- |
|  |  |  | x | y | z |  |  |
| Right I-IV | **Thalamus** | **--** | **14** | **-20** | **14** | **192** | **13.05** |
|  | Thalamus | -- | 12 | -30 | 10 | 192 | 10.53 |
|  | Hippocampus | -- | 22 | -34 | -2 | 192 | 9.40 |
|  | **Posterior Cingulate** | **30** | **-4** | **-48** | **22** | **229** | **11.35** |
|  | Precuneus | 23 | 6 | -48 | 24 | 229 | 10.39 |
|  | Cingulate gyrus | 23 | 0 | -26 | 28 | 229 | 10.23 |
| Right V | **Posterior Cingulate** | **23** | **-6** | **-50** | **22** | **210** | **11.08** |
|  | Precuneus | 23 | 6 | -48 | 26 | 210 | 10.66 |
|  | Posterior Cingulate | 23 | -6 | -38 | 28 | 210 | 10.10 |

**Supplementary Table 2.** MNI coordinates of the local maxima of brain regions showing significant functional connectivity with cerebellar seed regions in Parkinson’s patients OFF medication. Peak values for each cluster are shown in bold.

| **Seed** | **Region** | **BA** | **MNI coordinates** | | | **Cluster size** | **T-Value** |
| --- | --- | --- | --- | --- | --- | --- | --- |
|  |  |  | x | y | z |  |  |
| Right Crus I | **Precuneus** | **7** | **2** | **-68** | **40** | **1697** | **14.05** |
|  | Cuneus | 19 | -6 | -80 | 38 | 1697 | 11.61 |
|  | Cuneus | 19 | 12 | -70 | 40 | 1697 | 11.56 |
|  | **Caudate** | **--** | **16** | **16** | **16** | **102** | **11.63** |
|  | Caudate | -- | 20 | 8 | 20 | 102 | 7.91 |
|  | **Superior Frontal Gyrus** | **46** | **26** | **48** | **10** | **198** | **11.31** |
|  | Superior Frontal Gyrus | 46 | 24 | 48 | 18 | 198 | 11.01 |
|  | Middle Frontal Gyrus | 11 | 30 | 54 | 4 | 198 | 9.62 |
|  | **Superior Occipital Gyrus** | **18** | **-20** | **-100** | **16** | **392** | **11.13** |
|  | Middle Occipital | 18 | -30 | -94 | 6 | 392 | 10.60 |
|  | **Angular Gyrus** | **7** | **-36** | **-62** | **38** | **340** | **10.98** |
|  | Inferior Parietal Gyrus | 40 | -48 | -50 | 40 | 340 | 10.72 |
|  | Angular Gyrus | 39 | -46 | -60 | 28 | 340 | 9.26 |
|  | **Inferior Temporal Gyrus** | **20** | **-46** | **-26** | **-8** | **183** | **10.87** |
|  | Inferior Temporal Gyrus | 20 | -44 | -28 | -16 | 183 | 10.00 |
|  | Hippocampus | 20 | -36 | -22 | -14 | 183 | 8.73 |
|  | **Middle Temporal Gyrus** | **37** | **42** | **-62** | **12** | **127** | **10.84** |
|  | Middle Occipital Gyrus | 39 | 34 | -72 | 20 | 127 | 9.88 |
|  | Middle Occipital Gyrus | 39 | 38 | -76 | 30 | 127 | 9.70 |
|  | **Medial Superior Frontal Gyrus** | **9** | **4** | **52** | **44** | **119** | **10.54** |
|  | Medial Superior Frontal Gyrus | 8 | 0 | 40 | 52 | 119 | 10.53 |
|  | **Middle Frontal Gyrus** | **46** | **-26** | **48** | **32** | **367** | **10.49** |
|  | Middle Frontal Gyrus | 46 | -28 | 40 | 30 | 367 | 10.45 |
|  | Middle Frontal Gyrus | 46 | -40 | 42 | 24 | 367 | 10.00 |
|  | **Orbital Medial Frontal Cortex** | **11** | **-32** | **56** | **-6** | **153** | **9.48** |
|  | Orbital Superior Frontal Cortex | 11 | -26 | 56 | 0 | 153 | 9.28 |
|  | Middle Frontal Gyrus | 47 | -34 | 46 | 4 | 153 | 8.67 |
| Right Crus II | **Superior Frontal Gyrus** | **46** | **24** | **46** | **14** | **433** | **14.89** |
|  | Middle Frontal Gyrus | 46 | 44 | 52 | 8 | 433 | 10.79 |
|  | Orbital Superior Frontal Cortex | 11 | 22 | 58 | 0 | 433 | 9.31 |
|  | **Posterior Cingulate** | **23** | **-10** | **-50** | **28** | **1992** | **14.81** |
|  | Thalamus | -- | 6 | -20 | 12 | 1992 | 11.73 |
|  | Thalamus | -- | -2 | -18 | 10 | 1992 | 11.71 |
|  | **Fusiform Gyrus** | **20** | **-42** | **-26** | **-14** | **387** | **12.39** |
|  | Middle Temporal Gyrus | 20 | -60 | -18 | -22 | 387 | 10.70 |
|  | Middle Temporal Gyrus | 20 | -52 | -14 | -12 | 387 | 9.95 |
|  | **Orbital Medial Frontal Cortex** | **11** | **-4** | **50** | **-12** | **238** | **12.12** |
|  | Anterior Cingulate | 10 | -6 | 44 | -4 | 238 | 10.17 |
|  | Orbital Medial Frontal Cortex | 10 | 10 | 44 | -8 | 238 | 10.10 |
|  | **Hippocampus** | **--** | **40** | **-22** | **-16** | **507** | **11.73** |
|  | Inferior Temporal Gyrus | 20 | 62 | -28 | -16 | 507 | 11.03 |
|  | Inferior Temporal Gyrus | 20 | 52 | -14 | -24 | 507 | 10.81 |
|  | **Angular Gyrus** | **39** | **-48** | **-68** | **40** | **125** | **10.39** |
|  | Angular Gyrus | 39 | -44 | -60 | 26 | 125 | 9.34 |
|  | Angular Gyrus | 39 | -42 | -62 | 36 | 125 | 9.31 |
|  | **Caudate** | **--** | **-18** | **-10** | **22** | **113** | **9.84** |
| Right I-IV | **Inferior Parietal Gyrus** | **40** | **42** | **-48** | **44** | **688** | **13.15** |
|  | Inferior Parietal Gyrus | 40 | 48 | -54 | 50 | 688 | 11.05 |
|  | Angular Gyrus | 39 | 42 | -58 | 44 | 688 | 10.36 |
|  | **Inferior Temporal Gyrus** | **20** | **48** | **-38** | **-20** | **255** | **12.87** |
|  | Inferior Temporal Gyrus | 20 | 50 | -28 | -24 | 255 | 9.88 |
|  | **Angular Gyrus** | **39** | **-54** | **-66** | **34** | **339** | **11.80** |
|  | Angular Gyrus | 40 | -40 | -50 | 36 | 339 | 9.55 |
|  | Angular Gyrus | 39 | -44 | -58 | 44 | 339 | 9.50 |
|  | **Middle Frontal Gyrus** | **46** | **28** | **52** | **30** | **234** | **10.58** |
|  | Middle Frontal Gyrus | 44 | 40 | 24 | 38 | 234 | 10.44 |
|  | Middle Frontal Gyrus | 9 | 26 | 28 | 34 | 234 | 9.84 |
|  | **Middle Occipital Gyrus** | **19** | **-32** | **-86** | **34** | **102** | **9.84** |
|  | Superior Occipital Gyrus | 19 | -24 | -82 | 34 | 102 | 9.33 |
| Right V | **Inferior Parietal** **Gyrus** | **40** | **46** | **-54** | **42** | **1270** | **12.22** |
|  | Angular Gyrus | 7 | 34 | -68 | 54 | 1270 | 11.21 |
|  | Inferior Parietal Gyrus | 2 | 52 | -36 | 50 | 1270 | 10.40 |
|  | **Rostral Cingulate Zone** | **24** | **-8** | **34** | **14** | **222** | **11.46** |
|  | Rostral Cingulate Zone | 32 | -6 | 30 | 24 | 222 | 9.25 |
|  | Rostral Cingulate Zone | 32 | -6 | 42 | 16 | 222 | 9.25 |
|  | **Middle Temporal Gyrus** | **20** | **56** | **-26** | **-8** | **790** | **11.40** |
|  | Inferior Temporal Gyrus | 20 | 56 | -20 | -22 | 790 | 11.30 |
|  | **Middle Frontal Gyrus** | **46** | **-36** | **28** | **34** | **372** | **11.20** |
|  | Middle Frontal Gyrus | 9 | -28 | 32 | 46 | 372 | 11.20 |
|  | Inferior Frontal Gyrus | 48 | -46 | 24 | 26 | 372 | 9.89 |
|  | **Postcentral Gyrus** | **48** | **54** | **-20** | **36** | **138** | **9.26** |
|  | **Middle Occipital Gyrus** | **19** | **-32** | **-84** | **32** | **138** | **11.06** |
|  | Superior Occipital Gyrus | 19 | -24 | -84 | 36 | 138 | 9.68 |
|  | Superior Occipital Gyrus | 19 | -22 | -92 | 34 | 138 | 9.39 |
|  | **Rostral Cingulate Zone** | **32** | **6** | **40** | **18** | **131** | **9.38** |
|  | Rostral Cingulate Zone | 24 | 6 | 38 | 10 | 131 | 8.28 |
|  | **Middle Occipital Gyrus** | **19** | **-32** | **-68** | **36** | **274** | **9.76** |
|  | Angular Gyrus | 39 | -42 | -66 | 52 | 274 | 9.44 |
|  | Inferior Parietal Gyrus | 40 | -50 | -44 | 40 | 274 | 9.26 |
| Right VI | **Middle Frontal Gyrus** | **46** | **-36** | **42** | **26** | **277** | **11.48** |
|  | Middle Frontal Gyrus | 46 | -40 | 52 | 16 | 277 | 9.90 |
|  | Middle Frontal Gyrus | 46 | -28 | 40 | 32 | 277 | 9.09 |
|  | **Middle Frontal Gyrus** | **46** | **40** | **50** | **14** | **257** | **11.09** |
|  | Middle Frontal Gyrus | 10 | 34 | 60 | 8 | 257 | 10.43 |
|  | Middle Frontal Gyrus | 10 | 28 | 50 | 8 | 257 | 9.90 |
|  | **Superior Temporal Gyrus** | **48** | **-56** | **0** | **0** | **136** | **10.74** |
|  | Superior Temporal Gyrus | 48 | -52 | 12 | -2 | 136 | 10.25 |
|  | Inferior Frontal Gyrus | 38 | -48 | 18 | -10 | 136 | 8.38 |
|  | **Postcentral Gyrus** | **2** | **-40** | **-44** | **64** | **117** | **10.48** |
|  | Postcentral Gyrus | 2 | -56 | -26 | 50 | 117 | 9.80 |
|  | Postcentral Gyrus | 2 | -50 | -34 | 56 | 117 | 8.90 |
|  | **Rostral Cingulate Zone** | **32** | **-8** | **38** | **24** | **115** | **9.11** |
|  | Rostral Cingulate Zone | 32 | -8 | 32 | 16 | 115 | 9.04 |
| Right VIIIa | **Precuneus** | **5** | **0** | **-46** | **54** | **243** | **10.50** |
|  | Precuneus | 5 | 10 | -54 | 68 | 243 | 10.08 |
|  | Precuneus | 7 | -2 | -58 | 56 | 243 | 9.45 |

**Supplementary Table 3.** MNI coordinates of the local maxima of cerebellar regions showing significant functional connectivity with each cerebellar seed in Parkinson’s patients ON medication. Peak values for each cluster are shown in bold.

| **Seed** | **Region** | **MNI coordinates** | | | **Cluster size** | **T-Value** |
| --- | --- | --- | --- | --- | --- | --- |
|  |  | x | y | z |  |  |
| Right Crus I | **Right VI** | **14** | **-67** | **-12** | **1316** | **21.24** |
|  | Left V | -3 | -69 | -11 | 1316 | 13.03 |
|  | Right I-IV | 9 | -52 | -7 | 1316 | 10.79 |
|  | **Right VI** | **37** | **-63** | **-25** | **9935** | **21.22** |
|  | Right VI | 30 | -71 | -23 | 9935 | 19.62 |
|  | **Right VIIb** | **31** | **-59** | **-45** | **817** | **16.84** |
|  | Right Crus II | 35 | -68 | -48 | 817 | 10.87 |
|  | **Right IX** | **9** | **-55** | **-40** | **287** | **11.50** |
|  | Right IX | 9 | -47 | -46 | 287 | 10.64 |
|  | **Left Crus I** | **-27** | **-71** | **-28** | **313** | **11.05** |
|  | Left Crus I | -39 | -64 | -29 | 313 | 10.24 |
| Right Crus II | **Right Crus I** | **14** | **-79** | **-31** | **7798** | **17.64** |
|  | Right Crus I | 39 | -73 | -28 | 7798 | 16.55 |
|  | **Right IX** | **8** | **-51** | **-45** | **451** | **15.68** |
|  | **Left VI** | **-23** | **-68** | **-30** | **574** | **14.67** |
|  | Left VI | -28 | -58 | -33 | 574 | 10.37 |
|  | **Left Crus I** | **-10** | **-78** | **-25** | **498** | **12.98** |
|  | Left Crus II | -13 | -89 | -34 | 498 | 11.71 |
|  | Left Crus II | -10 | -80 | -38 | 498 | 9.25 |
|  | **Left Crus II** | **-26** | **-76** | **-42** | **330** | **12.72** |
|  | Left Crus I | -27 | -68 | -37 | 330 | 10.47 |
|  | Left Crus I | -34 | -64 | -38 | 330 | 9.19 |
|  | **Left IX** | **-4** | **-54** | **-45** | **224** | **12.38** |
|  | **Right VIIb** | **26** | **-66** | **-50** | **203** | **10.63** |
|  | Right VIIb | 22 | -72 | -54 | 203 | 9.11 |
|  | **Left Crus II** | **-15** | **-81** | **-50** | **133** | **10.39** |
| Right I-IV | **Left I-IV** | **-10** | **-39** | **-21** | **272950** | **25.42** |
|  | **Vermis VI** | **-2** | **-64** | **-29** | **232** | **14.85** |
|  | **Left Crus I** | **-11** | **-87** | **-28** | **128** | **11.00** |
| Right V | **Left Crus I** | **-29** | **-69** | **-28** | **441** | **11.32** |
|  | Left VI | -12 | -70 | -24 | 441 | 9.66 |
|  | **Left Crus I** | **-44** | **-67** | **-32** | **173** | **10.37** |
| Right VI | **Right V** | **24** | **-47** | **-23** | **23786** | **23.84** |
|  | **Vermis IX** | **-3** | **-58** | **-36** | **894** | **12.90** |
|  | Vermis VIIIa | 1 | -55 | -32 | 894 | 10.43 |
|  | Right IX | 7 | -52 | -36 | 894 | 9.61 |
|  | **Left IX** | **-13** | **-53** | **-51** | **108** | **12.56** |
|  | **Left Crus I** | **-38** | **-66** | **-35** | **190** | **12.32** |
|  | Left Crus I | -37 | -76 | -34 | 190 | 10.54 |
|  | **Left Crus I** | **-11** | **-77** | **-27** | **158** | **11.40** |
| Right VIIIa | **Right VIIIb** | **27** | **-44** | **-45** | **2334** | **7.13** |
| Right VIIIb | **Left VIIIb** | **-15** | **-45** | **-54** | **491** | **13.02** |
|  | Left VIIIb | -24 | -43 | -53 | 491 | 11.20 |

**Supplementary Table 4**. MNI coordinates of the local maxima of cerebellar regions showing significant functional connectivity with each cerebellar seed in Parkinson’s patients OFF medication. Peak values for each cluster are shown in bold.

| **Seed** | **Region** | **MNI coordinates** | | | **Cluster size** | **T-Value** |
| --- | --- | --- | --- | --- | --- | --- |
|  |  | x | y | z |  |  |
| Right Crus I | **Right VI** | **17** | **-74** | **-25** | **411260** | **25.26** |
|  | **Left V** | **-19** | **-48** | **-24** | **483** | **13.79** |
|  | **Right I-IV** | **1** | **-51** | **-1** | **423** | **12.30** |
|  | **Left IX** | **-10** | **-52** | **-45** | **847** | **12.13** |
|  | Left VIIIb | -17 | -44 | -52 | 847 | 10.34 |
| Right Crus II | **Right Crus I** | **42** | **-68** | **-25** | **419870** | **21.77** |
|  | **Left IX** | **-10** | **-54** | **-44** | **847** | **15.62** |
|  | Left IX | -13 | -47 | -52 | 847 | 10.87 |
|  | **Right X** | **26** | **-38** | **-40** | **409** | **13.34** |
|  | Right X | 20 | -40 | -47 | 409 | 10.40 |
| Right I-IV | **Right V** | **25** | **-40** | **-27** | **330810** | **21.89** |
| Right V | **Left Crus I** | **-42** | **-73** | **-31** | **107** | **11.66** |
| Right VI | **Left Crus II** | **-32** | **-63** | **-46** | **11599** | **13.24** |
|  | Left VIIb | -24 | -66 | -48 | 11599 | 11.36 |
|  | Left VIIb | -28 | -72 | -53 | 11599 | 11.13 |
|  | **Right Crus I** | **42** | **-75** | **-34** | **111** | **10.86** |
|  | **Vermis IX** | **2** | **-61** | **-46** | **156** | **10.85** |
|  | Right IX | 6 | -56 | -53 | 156 | 10.05 |
| Right VIIIa | **Right VI** | **16** | **-67** | **-23** | **2543** | **12.54** |
|  | Right VI | 31 | -58 | -31 | 2543 | 12.07 |
|  | Right VI | 20 | -60 | -24 | 2543 | 10.98 |
|  | **Right Crus I** | **46** | **-60** | **-31** | **104** | **12.47** |
|  | **Left VI** | **-30** | **-52** | **-30** | **619** | **12.43** |
|  | Left Crus I | -37 | -62 | -30 | 619 | 10.20 |
|  | **Left VIIIb** | **-21** | **-51** | **-57** | **194** | **12.29** |
|  | Left VIIIb | -26 | -45 | -52 | 194 | 10.39 |
|  | Left VIIIa | -34 | -49 | -50 | 194 | 10.33 |
|  | Left VIIIa | **-7** | **-67** | **-47** | **221** | **12.17** |
| Right VIIIb | **Right IX** | **6** | **-61** | **-47** | **3725** | **14.63** |
|  | **Right VI** | **10** | **-63** | **-23** | **565** | **20.70** |
|  | Right V | 10 | -54 | -19 | 565 | 10.24 |
|  | **Left VIIIb** | **-15** | **-46** | **-59** | **1944** | **15.53** |
|  | Left VIIIb | -24 | -49 | -56 | 1944 | 12.43 |
|  | Left VIIIb | -14 | -57 | -59 | 1944 | 12.19 |
|  | **Left VI** | **-6** | **-68** | **-22** | **210** | **12.03** |
|  | Left V | -2 | -58 | -23 | 210 | 10.84 |
